# Diffuse hepatic artery dilation: an uncommon manifestation of early cirrhosis

**DOI:** 10.1093/omcr/omaf245

**Published:** 2025-11-26

**Authors:** Van Trung Hoang, Hoang Anh Thi Van, The Huan Hoang, Vichit Chansomphou

**Affiliations:** Department of Radiology, Thien Hanh Hospital, 17 Nguyen Chi Thanh Street, Dak Lak 63000, Vietnam; Department of Radiology, Thien Hanh Hospital, 17 Nguyen Chi Thanh Street, Dak Lak 63000, Vietnam; Department of Radiology, Thien Hanh Hospital, 17 Nguyen Chi Thanh Street, Dak Lak 63000, Vietnam; Department of Radiology, Savannakhet Medical-Diagnostic Center, 266/5 Chaimeuang Phetsarat Street, Kaysone Phomvihane 13000, Lao People’s Democratic Republic

**Keywords:** cirrhosis, CT, hepatic artery dilation, portal hypertension, visceral vessels, vascular anomalies

## Abstract

We report a case of a 65-year-old man with diffuse hepatic artery dilation. Laboratory tests and imaging studies indicated signs of chronic liver disease. Specific measurements of dilated vessels are provided, and relevant diagnostic and therapeutic considerations are discussed. The patient was treated with common hepatoprotective agents and the progression of cirrhosis was prevented. Importantly, this case emphasizes the need to distinguish hepatic artery dilation from other vascular anomalies, particularly in the absence of portal hypertension, through high-resolution imaging and clinical correlation.

We present the case of a 65-year-old male patient with an unremarkable personal and family medical history, who presented for a routine physical examination. His vital signs were within normal limits: blood pressure was 130/70 mmHg, pulse rate was 80 bpm, respiratory rate was 22 breaths per minute, and temperature was 37°C.

Liver function tests revealed aspartate aminotransferase (AST) of 67 IU/L (normal range, 0–40), alanine aminotransferase (ALT) of 85 IU/L (normal range, 0–40), and gamma-glutamyl transpeptidase (GGT) of 126 IU/L (normal range, 12–64). Tests for hepatitis viruses were negative, and both bilirubin levels and alpha-fetoprotein were within normal ranges.

An ultrasound examination revealed diffuse dilatation of the celiac trunk and hepatic arteries, alongside mild liver surface irregularities and increased perfusion of the liver parenchyma. Liver elastography showed liver fibrosis at F2 level according to Metavir classification (Fig. 1). A computed tomography (CT) scan of the abdomen, performed with a 1.25 mm slice thickness in the arterial phase, confirmed diffuse dilatation of the celiac trunk and hepatic arteries. The celiac trunk measured approximately 12 mm in diameter (normal range: 6–7 mm), the common hepatic artery measured 14 mm (normal range: 4–5 mm), and the splenic artery measured 5 mm (normal range: 4–5 mm) (Fig. 2). No abnormal shunts, signs of portal hypertension, masses, or aneurysms were detected.

**Figure 1:**
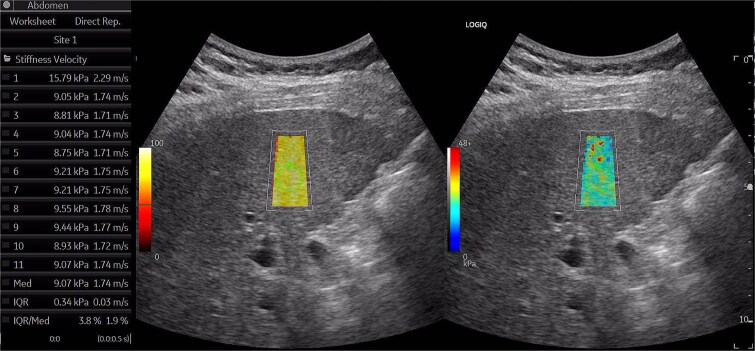
Ultrasound images show a slightly coarse liver texture and a mildly obtuse liver angle. Liver elasticity, assessed with shear wave Elastography on the GE LOGIQ FORTIS machine, corresponds to F2 according to the Metavir classification, with a median velocity of approximately 1.75 m/s. the liver stiffness classification table for the GE LOGIQ FORTIS machine is as follows: F0 (<1.35 m/s), F1 (1.35–1.65 m/s), F2 (1.66–1.76 m/s), F3 (1.77–1.99 m/s), F4 (>1.99 m/s).

**Figure 2:**
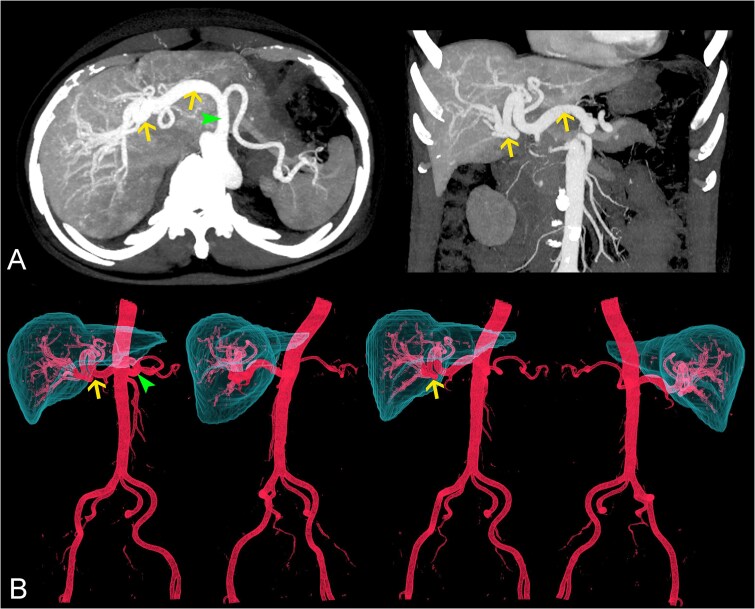
(A) Axial and coronal MIP and (B) 3D-reconstruction CT images showing diffuse dilatation of the celiac trunk (arrowheads) and hepatic arteries (arrows). Note the absence of abnormal shunts, signs of portal hypertension, masses, and aneurysms. The splenic artery showed normal size.

These findings ruled out other vascular anomalies such as aneurysms, arteriovenous malformations, or pseudoaneurysms, which may exhibit focal dilations or turbulent flow on Doppler imaging. By contrast, the diffuse, symmetric, and stable nature of the dilation in our case suggested a compensatory vascular response associated with early cirrhotic changes rather than a true vascular malformation.

He was advised to make dietary adjustments and was administered hepatoprotective agents at intermittent intervals. After five weeks of treatment, liver function test parameters normalized. The patient was followed up for two years, during which his laboratory results remained within normal limits. Serial hepatobiliary ultrasound images showed that the hepatic artery dilation remained stable over time. The final diagnosis was hepatic artery ectasia with early cirrhosis.

Currently, no specific diagnostic standard for hepatic artery dilation related to early cirrhosis has been established. To accurately diagnose hepatic artery dilatation, it is necessary to determine the percentile value of the hepatic artery diameter of that population by age [1, 2]. However, there have been no large studies determining this value in the general population with our patient. From a comparative perspective, the most common cause of hepatic artery dilation is secondary to portal hypertension, typically presenting with tortuous ‘corkscrew’ morphology, whereas vascular malformations (e.g. Osler-Weber-Rendu disease) or diffuse hemangiomatosis are much rarer. In contrast, the uniform, non-turbulent dilation observed here, together with early cirrhotic changes, indicates a distinct pathophysiological mechanism.

This highlights the importance of differentiating true pathological arterial dilation from anatomical variants or compensatory changes in liver vasculature. For example, the ‘corkscrew’ appearance of the hepatic artery in advanced portal hypertension is typically associated with hemodynamic changes, unlike the uniform dilation seen here.

Further studies are needed to define clear diagnostic criteria. The imaging technique used included contrast-enhanced CT during the arterial phase with slice thicknesses of 1.25 mm, which provided high-resolution visualization of the dilated vessels [3, 4].

It is known that early cirrhosis leads to changes in the extracellular matrix and increased liver stiffness. If elastography (via Vibration-Controlled Transient Elastography or Shear Wave Elastography) or magnetic resonance elastography had been performed, we would have anticipated increased liver stiffness consistent with cirrhotic changes. Further assessment using these tools could provide additional insights into the degree of fibrosis present [5, 6].

The regulation of hepatic artery and portal vein flow is not fully elucidated at present. However, the mechanism underlying this dilation of the hepatic artery is related to structural alteration of the vessel wall itself (remodeling) and overexpression of vasodilators such as nitric oxide and adenosine. In the early stages of cirrhosis, hepatic artery dilation occurs despite normal portal resistance and is thought to be due to nitric oxide-mediated hypoxia in the liver. These lead to increased hepatic arterial circulation and dilation of the hepatic arteries [1, 2]. The most common cause of hepatic artery dilation is secondary to portal hypertension and cirrhosis. In portal hypertension, dilatation and spiraling of the hepatic artery (corkscrew appearance) is typical [2, 3]. Osler-Weber-Rendu disease is another rare cause of hepatic artery dilation. Arterial dilation can be external or intrahepatic and sometimes both [3, 4]. The hepatic artery may be dilated in patients with diffuse hepatic hemangiomatosis. This condition is rare and is usually found in neonates with abdominal distention and sometimes congestive heart failure. It usually involves skin involvement and individual forms are rare [1–4, 6].

Treatment for this condition largely focuses on managing the underlying liver disease and cirrhosis, with no specific therapy targeting the arterial dilation itself. In this case, dietary modifications and hepatoprotective agents helped stabilize the patient’s condition, preventing progression of cirrhosis. Further studies are needed to better understand the optimal management strategies for patients with hepatic artery dilation secondary to early cirrhosis. This emphasizes that management should prioritize the underlying hepatic disease rather than the vascular abnormality alone.

The patient reported no significant alcohol consumption, smoking, or other lifestyle factors that would adversely affect liver health. He had no known metabolic or cardiovascular comorbidities that could have contributed to his liver disease, and his physical activity levels were moderate. His diet, while not strictly monitored, was typical for his age group and region, with no extreme risk factors.

In this case, differentiation from other vascular anomalies was accomplished based on several features: absence of signs of portal hypertension, lack of turbulent flow or pseudoaneurysm formation, and the consistency of the dilation over time on serial imaging. Furthermore, the presence of early cirrhotic changes on elastography and laboratory tests supported a diagnosis of hepatic artery ectasia rather than another vascular malformation.
